# Supplementary material for: Transcriptomic Insights into Mechanisms of Early Seed Maturation in the Garden Pea (Pisum sativum L.)
Source: Cells. 2020 Mar 23;9(3):779. doi: 10.3390/cells9030779 (PMC7140803; doi:10.3390/cells9030779)
Supplement: Supplementary file 1 [file cells-09-00779-s001.zip › Supplementary materials/FileS3.zip!/FileS2/Hormones - Results/Sample Similarity Analysis/Component Analysis.pdf]

Independent Component Analysis on module data (dmap)

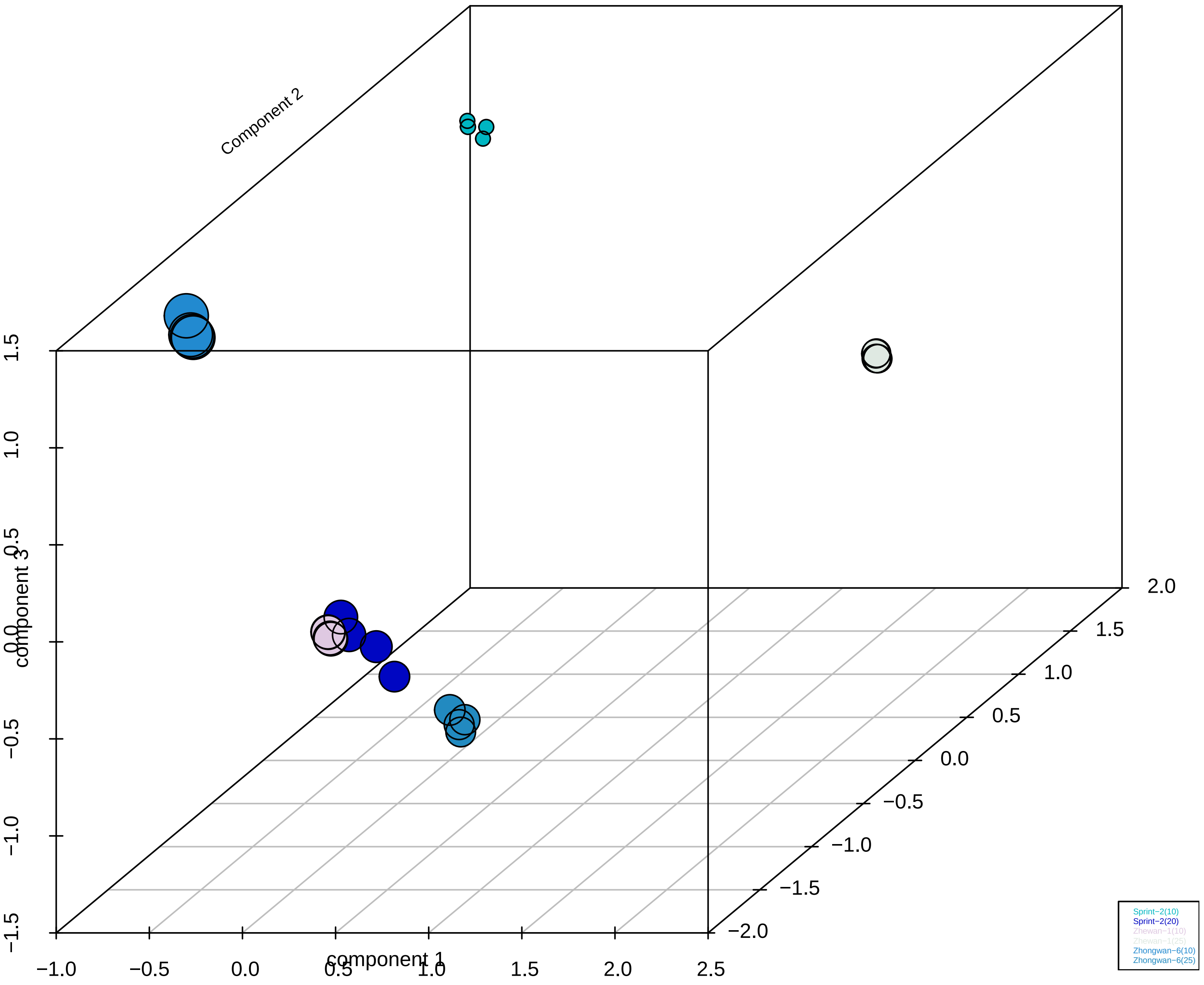

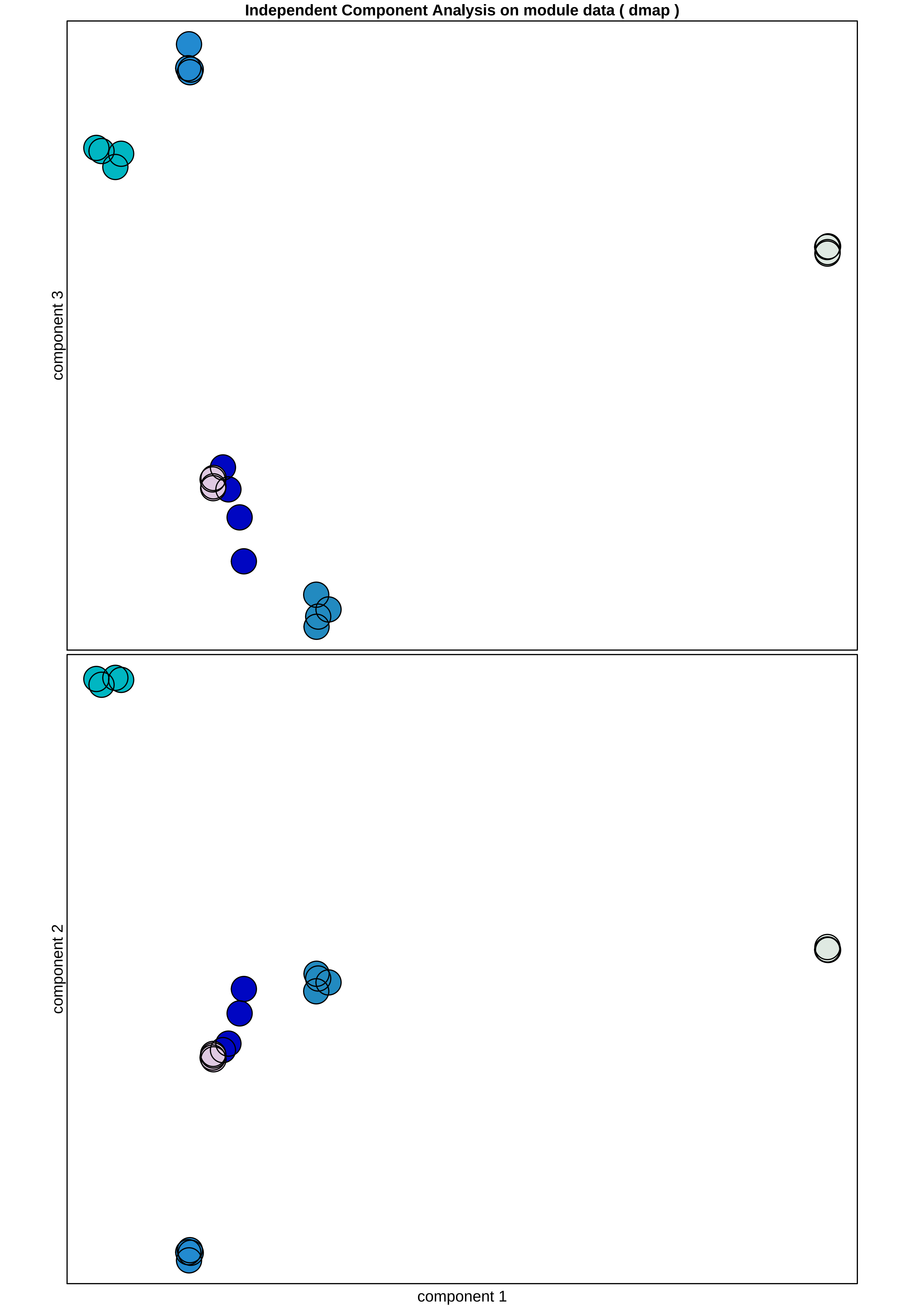
Independent Component Analysis on module data (dmap)
component 3
component 2
component 1

Independent Component Analysis on module data (dmap)

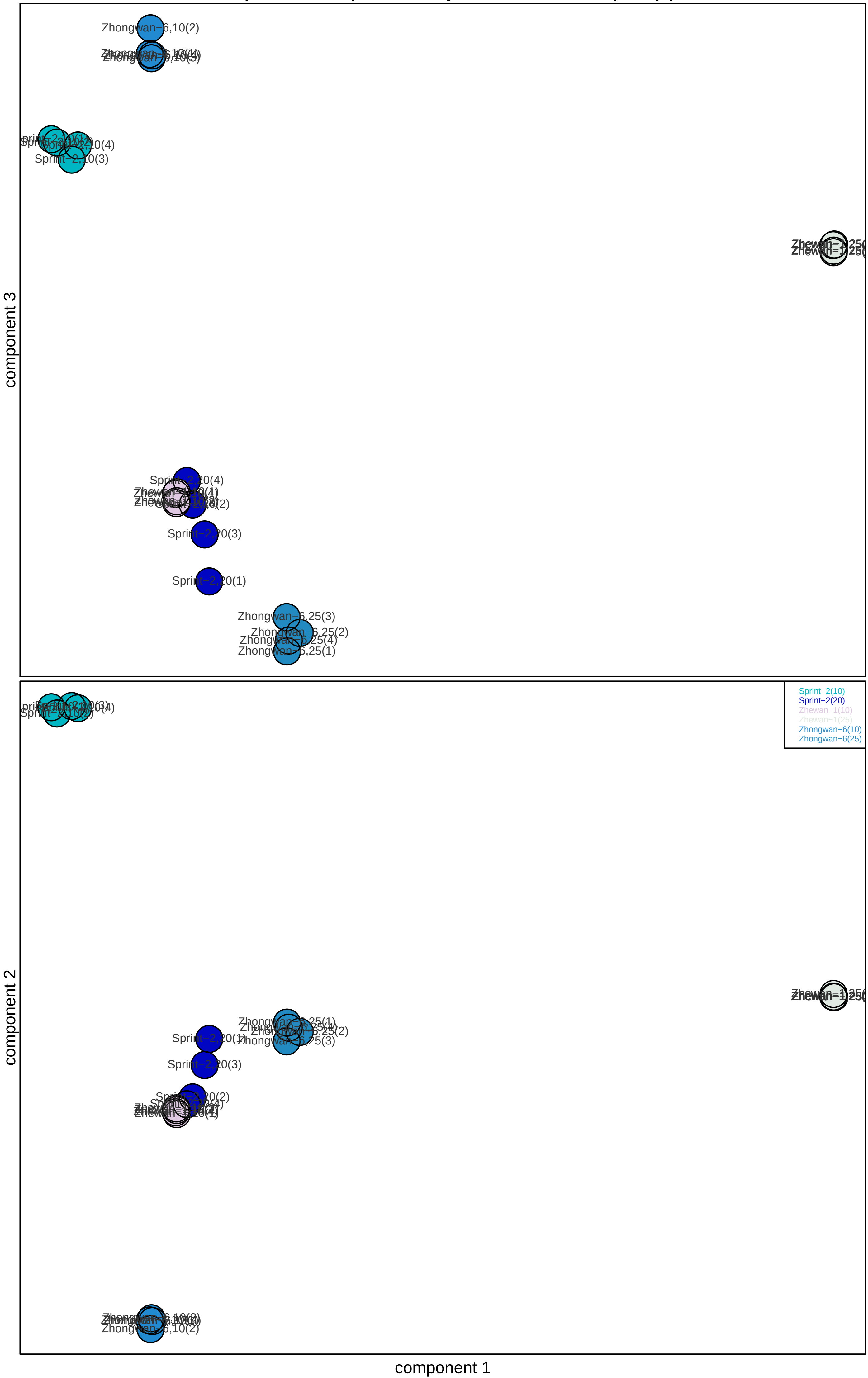

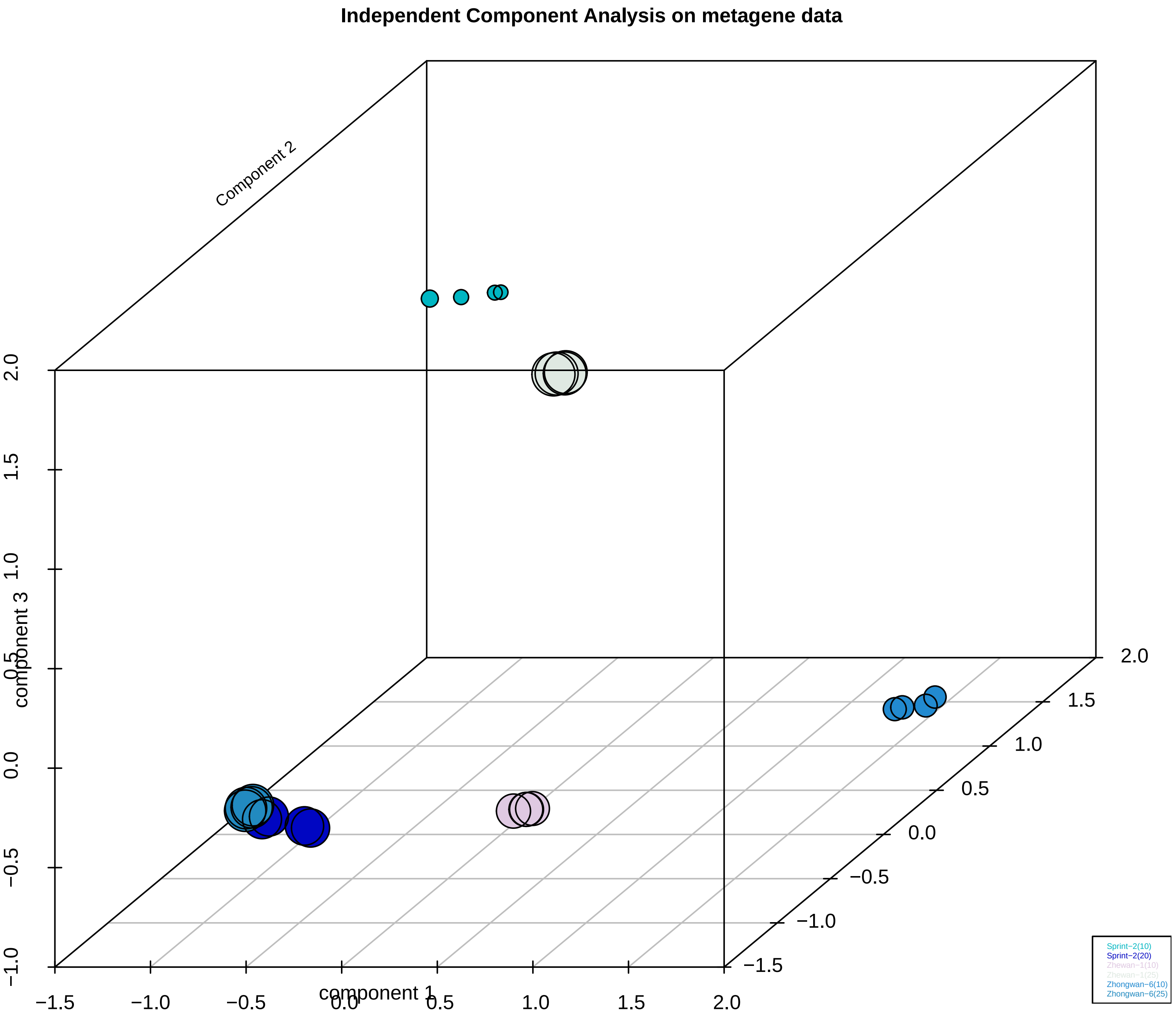
Independent Component Analysis on metagene data
Component 2
component 3
component 1
2.0
1.5
1.0
0.5
0.0
−0.5
−1.0
−1.5
−1.0
−0.5
0.0
0.5
1.0
1.5
2.0
−1.5
−1.0
−0.5
0.0
0.5
1.0
1.5
2.0
Sprint−2(10)
Sprint−2(20)
Zhewan−1(10)
Zhewan−1(25)
Zhongwan−6(10)
Zhongwan−6(25)

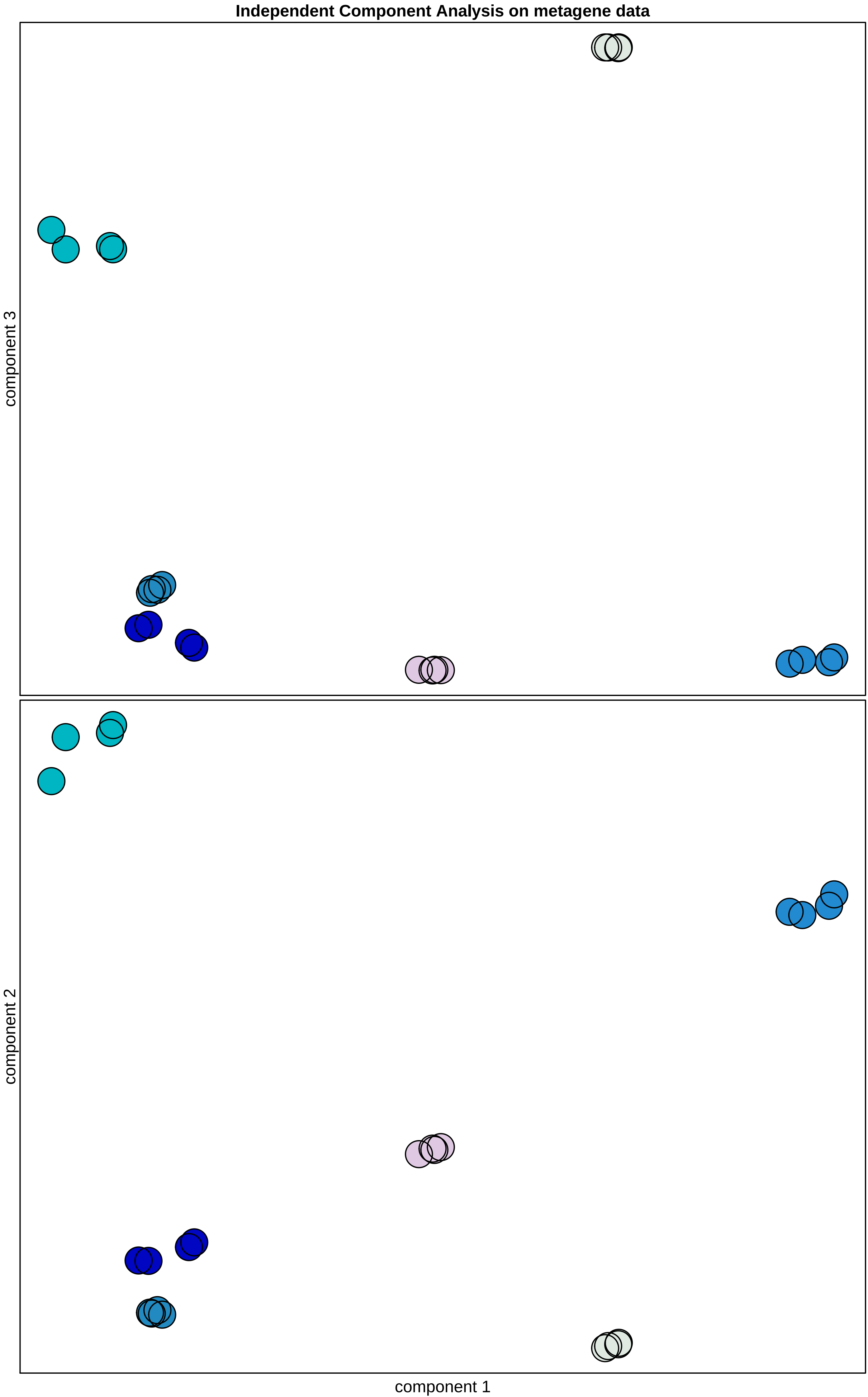
Independent Component Analysis on metagene data
component 3
component 2
component 1

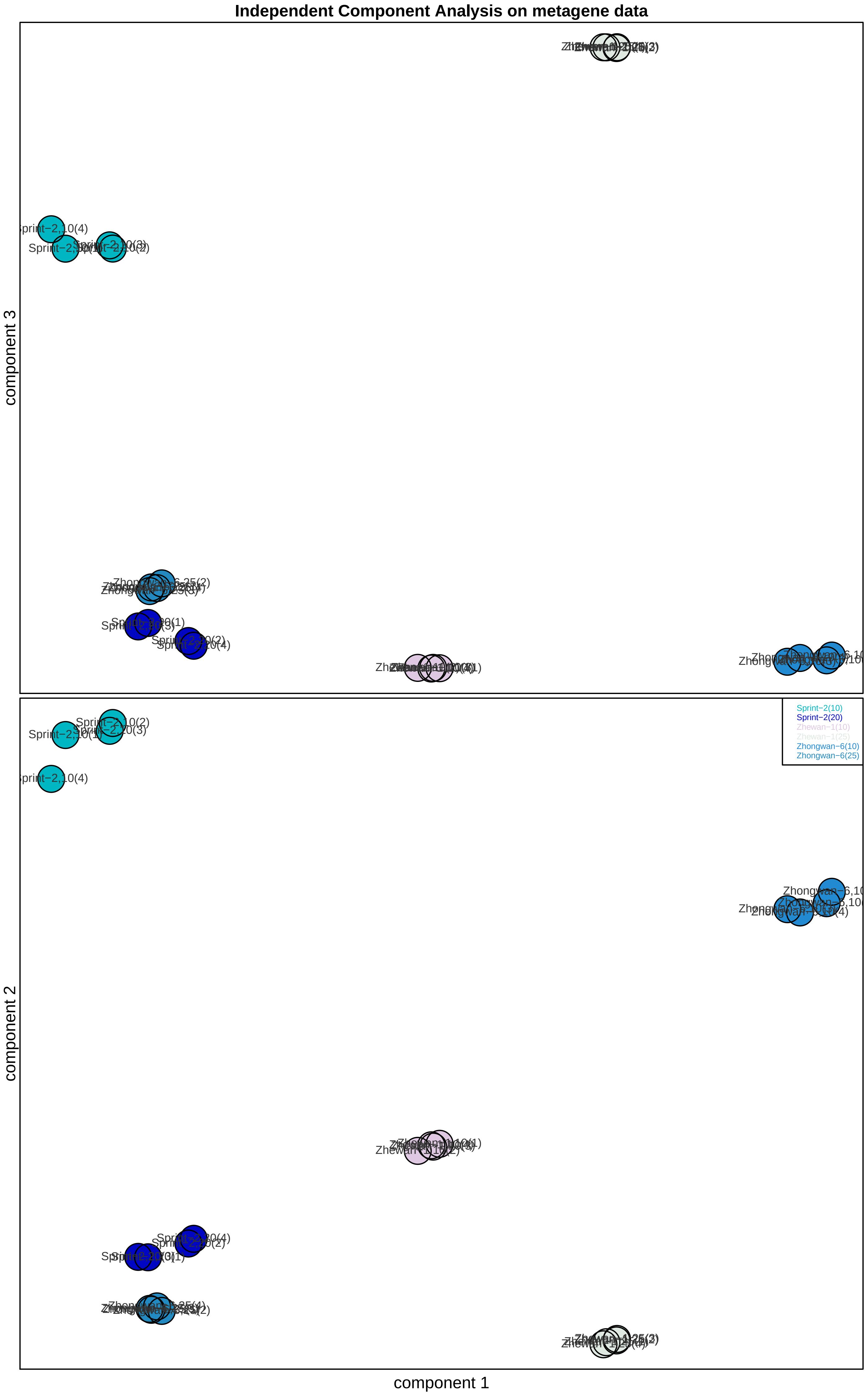
Independent Component Analysis on metagene data
component 3
component 2
component 1
Sprint−2(10)
Sprint−2(20)
Zhewan−1(10)
Zhewan−1(25)
Zhongwan−6(10)
Zhongwan−6(25)
